# Mendelian Randomization Studies of Major Depressive Disorder, Schizophrenia, Bipolar Disorder, and Cancers: A PRISMA Review and Meta-Analysis

**DOI:** 10.2174/0118715303432589260216095347

**Published:** 2026-04-08

**Authors:** Elsa Vitale, Lorenza Maistrello, Alessandro Rizzo, Matteo Santoni, Mohadeseh Motamed- Jahromi, Alessandra Sallustio, Francesco Tramacere

**Affiliations:** 1 Directorate of Health Professions and Nursing, ASL Bari, Bari, Italy;; 2 IRCCS San Camillo Hospital, Venice, Italy;; 3 S.S.D. C.O.r.O. Bed Management Presa in Carico, TDM, IRCCS Istituto Tumori “Giovanni Paolo II”, Viale Orazio Flacco 65, Bari, 70124, Italy;; 4 Oncology Unit, Macerata Hospital, Macerata, Italy;; 5 Department of Public Health, School of Health, Fasa University of Medical Sciences, Fasa, Iran;; 6 Radiotherapy Ward, Perrino Hospital, ASL Brindisi, Brindisi, Italy

**Keywords:** Psychiatric disorders, Mendelian randomization analysis, mental disorders, neoplasms, schizophrenia, bipolar disorder

## Abstract

**Introduction:**

Previous literature has reported ambiguous results regarding the association of severe psychiatric disorders and an increasing risk of cancer disease. The aim of our analysis was to explore any associations between severe psychiatric disorder (SPD), like schizophrenia (SCZ), Bipolar Disorder (BD) and recurrent major depression (MDD), and cancer diseases by meta-analyzing Mendelian Randomization (MR) studies available in the current literature.

**Methods:**

The present systematic review and meta-analysis was registered in the Prospero register (id. no. CRD420251072022). The databases consulted were Embase, Scopus, PubMed, and Web of Science. This study considered only studies that used MR to explore the causal effects of MDD, SCZ, and BD on cancer outcomes and that included MR as part of their analysis in humans.

**Results:**

A total of 7 MDD studies were selected for quantitative analysis. The findings of this study highlighted a statistically significant association between cancer and SCZ using the IVW method (OR = 1.05; 95% CI: [1.004; 1.105]; *P*= 0.032). Conversely, using the other two MR methods (MR-Egger and MR-Weighted Median) yielded no significant association, also in SCZ.

**Discussion:**

Literature on this topic remains scarce, and more studies are needed to assess the association between SPD and cancer.

**Conclusion:**

The need to improve health management strategies for individuals with SPDs remains unmet. Further investigation into biological functions and interdisciplinary associations will inform future interventions and public health policies to moderate cancer risk in this susceptible population.

## 1 INTRODUCTION

### 1.1 Severe Psychiatric Disorders and Cancer

The incidence of Severe Psychiatric Disorders (SPD), like schizophrenia (SCZ), Bipolar Disorder (BD), and recurrent major depression (MDD), is increasing in Western countries [1]. SPD has been linked to an increased risk of developing comorbid chronic conditions, including neurological and other mental as well as physical health problems, such as cancer [1, 2]. In this regard, literature has just suggested that people suffering from SCZ and BD may report a 2–3 times higher risk of chronic illnesses compared to the general population [3-5]. A previous systematic review and meta-analysis including 19 studies from Europe, Australia, North America, and East Asia reported that people suffering from SCZ recorded a 40% higher risk of cancer mortality compared to the general population, also considering a few important gender related covariates [6]. Additionally, SPD may have a significant impact on disability [7]. On the other hand, due to their SPD^,^ patients can frequently face several barriers to adequate cancer care that may increase cancer-related mortality [8, 9].

Very often, SPD is associated with several significant complications, such as stigma and discrimination, difficulties at work, a negative consequence both in an individual’s and family’s quality of life, reporting a higher burden [10] with also physical health problems, such as cardiovascular illness, infectious diseases, and cancer [11, 12]. Moreover, literature has reported ambiguous results regarding the association of SPD with the increasing risk of cancer disease, since there is evidence suggesting decreased [13, 14], and conversely, increased [9, 14] rates of cancers among these patients. In this direction, there is evidence suggesting a possible genetic association between SPD and cancer diseases [15, 16] and the other counterpart, which supports the absence of this association [17, 18]; in fact, confounding factors due to differences in diagnostic criteria, restricted sample sizes, and heterogeneity should be considered. Additionally, observational studies are exposed to confounding variables, such as smoking [19], alcohol use [20], physical activity [21], obesity [22], diabetes [23], and antipsychotic use [24].

Current genetic and molecular evidence suggests an involvement of SPDs in cancer pathogenesis; however, the exact linkage between SPDs and cancer still remains unknown. Genome-Wide Association Studies (GWASs) have identified a comprehensive spectrum of genetic variants across the entire genome associated with severe diseases [25]. Moreover, GWAS data can also be used to perform Mendelian Randomization (MR) analyses, which allow assessment of the direction of causal effects [26].

### 1.2 Mendelian Randomization Approach

Mendelian Randomization (MR) is a methodological approach that uses genetic variants as Instrumental Variables (IVs) to assess causal associations, free from confounding and reverse causality [27]. MR is also recognized as “Nature’s Randomized Trials” [28], which enhances traditional epidemiological methods by leveraging Mendel’s Second Law, which states that alleles are randomly allocated during gamete formation, leading to a randomized assignment of exposures [29]. The level of evidence of MR is between observational studies and randomized controlled trials. Well-designed MR studies can yield more reliable evidence than observational studies, providing several inputs for public health interventions, especially when randomized controlled trials are not practicable [30]. This allocation often takes place independently of environmental risk factors and introduces the onset of diseases and related risk factors. Two-step MR, a variant of MR, explores possible mediator effects between exposure and outcomes using a two-sample MR approach [31].

In the MR analysis, three main statistical models were adopted: the inverse-variance weighting (IVW) approach [32], the MR-Egger regression [33], and the weighted median approach [34].

The IVW methodology is preferred when all instrumental variables (IVs) are valid and do not exhibit pleiotropy [35].

The MR-Egger regression identifies and adjusts for pleiotropy in MR analyses, thereby mitigating the need for no pleiotropy between genetic variants within IVW methodologies. The intercept of the MR-Egger regression estimates the mean pleiotropic effect for each genetic variant [36].

The weighted median approach assumes that the medians are derived from weight-dependent sorting of all independent values within the SNP effect. The weighted median can be a robust estimate if the MR core hypotheses are satisfied with at least 50% genetic variation [37].

### 1.3 Aim

To explore any associations between SPDs and cancer diseases by meta-analyzing MR studies available in the current literature.

## 2 MATERIALS AND METHODS

### 2.1 Databases Explored

Embase

Scopus

PubMed

Web of Science

### 2.2 Mesh Terms Combined

Mesh terms combined with AND/OR Boolean terms were: “Basic Characteristics” AND “Mental Disorders” AND “Neoplasms” (Supplementary File **1**).

Supplementary File **1**: Search strings were developed to conduct this systematic and meta-analysis study.

### 2.3 Inclusion and Exclusion Criteria

The present study only includes studies with the following features:

- Studies that used MR to explore the causal relationships between MDD, SCZ, BD, and cancer.
- Studies that used genetic variation as an exposure to make causal inferences about cancer outcomes.
- Association studies that included MR in their analysis.
- Only studies including human beings.
- Only studies available in their full text versions and written in the English language.

### 2.4 Data Screening

The present systematic review and meta-analysis were registered in the PROSPERO register with ID no. CRD420251072022.

Initially, records were identified through a systematic database search and uploaded to a reference management software, where duplicates were removed. Then, two independent reviewers (E.V. & L.M.) assessed the titles and abstracts of the identified studies for inclusion, and unsuitable reports were removed. After that, articles were uploaded, and the full text was assessed more closely for eligibility.

**Fig. (1):**
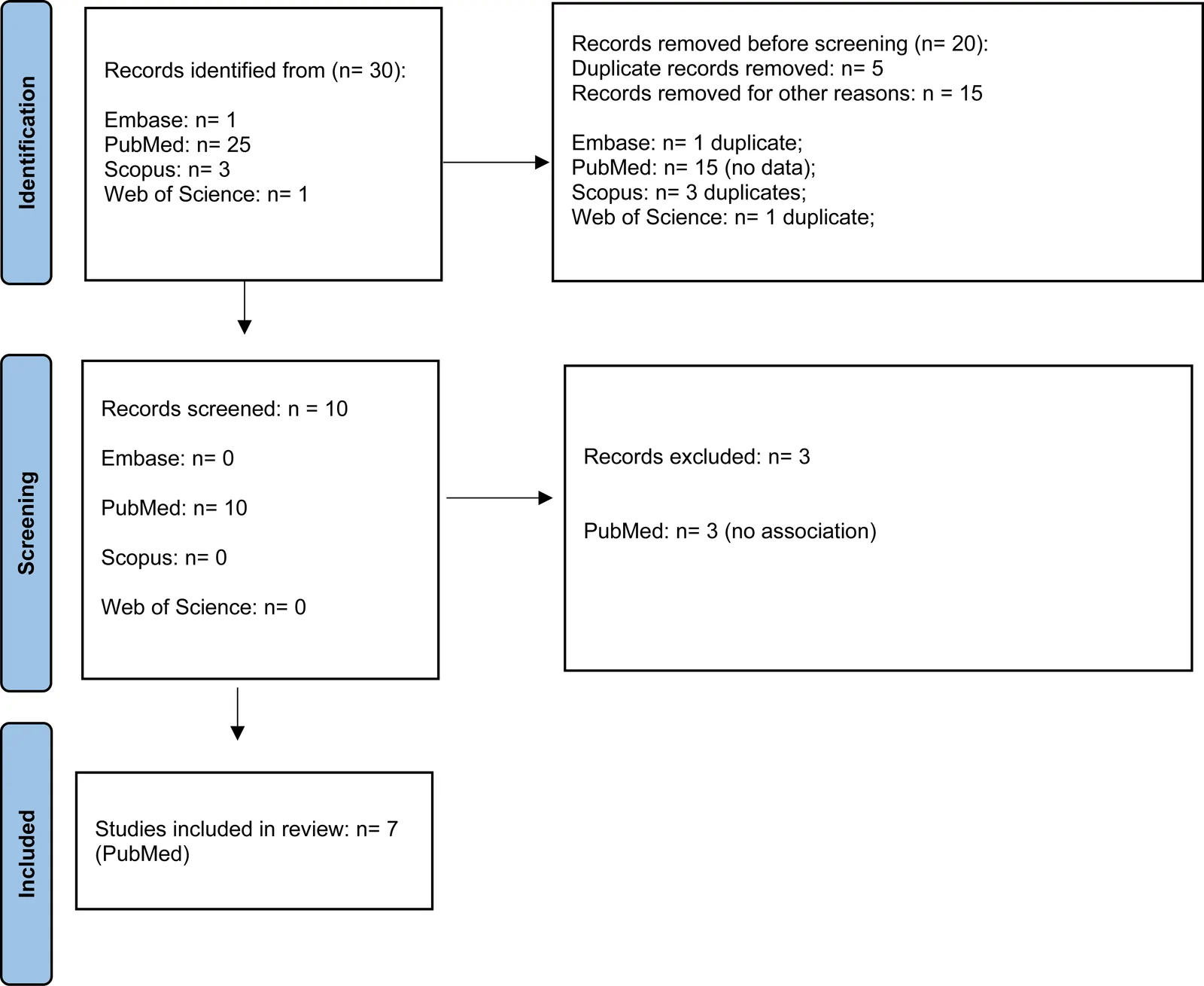
The PRISMA flow chart.

 Disagreements over whether a study should be included were resolved through discussion and consensus. If the disagreement remains, arbitration from another reviewer was provided. Data collection was extracted by considering study characteristics, participants, and incidence of cases of mental disorders, like Major Depressive Disorder, Schizophrenia, Bipolar Disorder, and cancer diseases (Fig. **1**).

### 2.5 Risk of Bias (Quality) Assessment

**Fig. (2):**
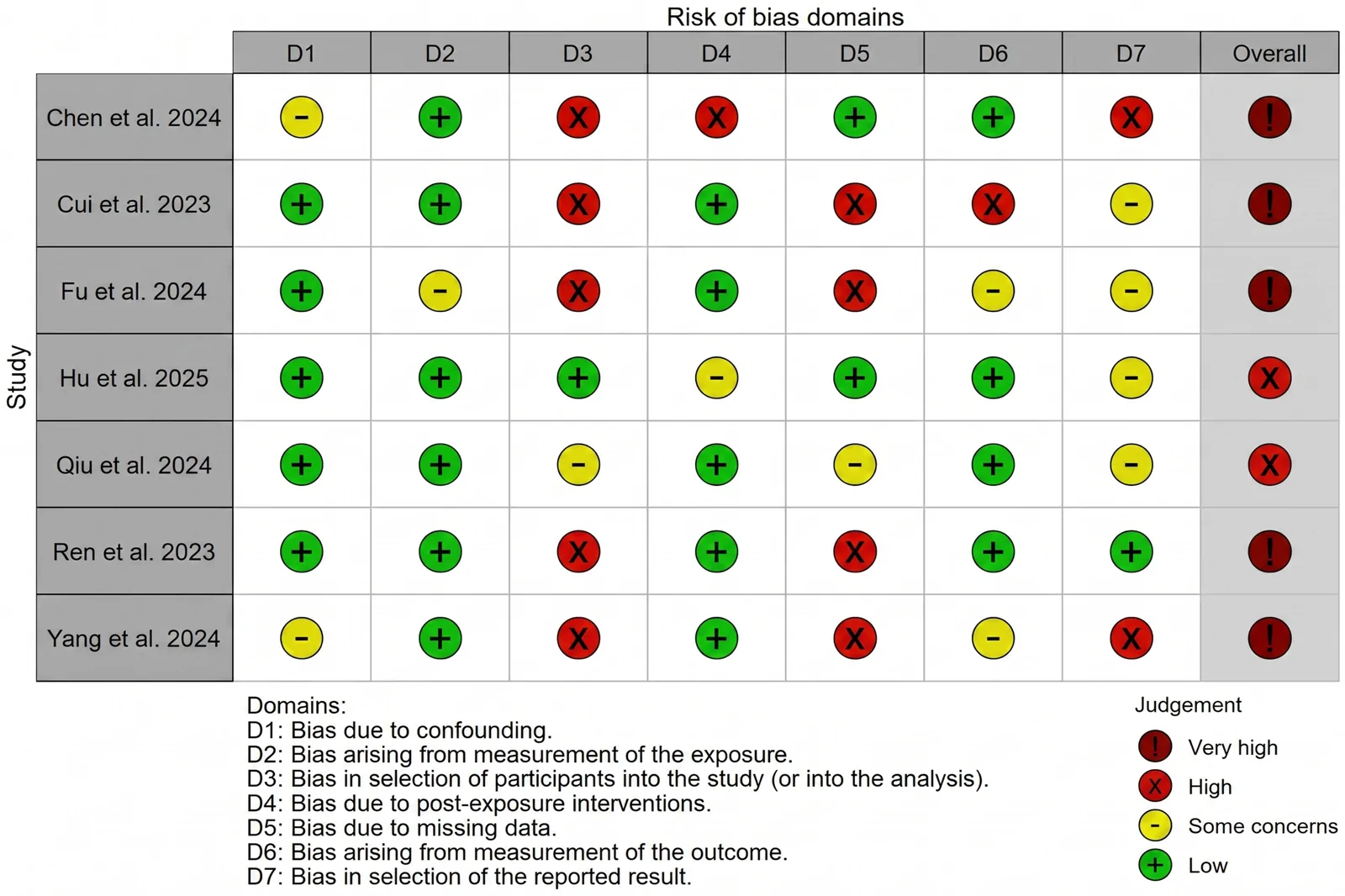
Risk bias assessment of the selected studies.

In observational studies, the presence of Risk of Bias was investigated using the risk of bias of exposures (ROBINS-E) tool [38]. The tool consisted of seven domains through which the presence of bias in the results could be investigated. These domains were identified based on empirical evidence and theoretical considerations and cover all types of bias that could influence the results of observational studies of exposures (Fig. **2**). The seven areas were:

(1) Bias due to confounding;

(2) Bias arising from measurement of the exposure;

(3) Bias in the selection of participants into the study (or into the analysis);

(4) Bias due to post-exposure interventions;

(5) Bias due to missing data;

(6) Bias arising from measurement of the outcome;

(7) Bias in the selection of the reported result.

### 2.6 Strategy for Data Synthesis

Study quality was assessed according to the recommendations outlined in the study protocol. Data extracted from the final set of included studies were synthesized both narratively and in tabular form. Statistical analyses were conducted using RStudio (version 4.5).

Three separate meta-analyses were performed to evaluate the association between genetic variants and three distinct mental illnesses: MDD, SCZ, and BD. For each condition, studies employing three MR methods were included: IVW, MR-Egger regression, and the Weighted Median approach. From each study, effect estimates were extracted as Odds Ratios (ORs) and their corresponding 95% confidence intervals (95% CI) for each MR method.

In total, nine separate meta-analyses were conducted - one for each MR method within each of the three psychiatric disorders under investigation. For each meta-analysis, the natural logarithm of the odds ratio (log(OR)) and the 95% CI bounds were calculated.

Heterogeneity across studies was assessed using Cochrane’s Q test. A statistically significant Q statistic (*P* < 0.05) was considered indicative of heterogeneity. To further quantify heterogeneity, the Tau-squared (τ^2^) statistic and the I^2^ index were calculated. I^2^ values of <25%, 25–50%, 50-75%, and >75% were interpreted as indicators of low, moderate, high, and very high heterogeneity, respectively [39]. Given that I^2^ can be unstable in meta-analyses with a limited number of studies, 95% confidence intervals for I^2^ were also reported to aid interpretation.

Random-effects meta-analyses were performed using the generic inverse-variance method. Forest plots were generated for each meta-analysis to visually present the pooled effect estimates and study-level data [40]. Publication bias was assessed through contour-enhanced funnel plots and formally tested using Egger’s regression test [41].

## 3 RESULTS

### 3.1 Study Characteristics

A total of 7 studies [42-48] on associations between SPD and cancer were considered for this analysis (Table **1**).

### 3.2 Major Depressive Disorder Associated with Cancers

A total of 7 studies for Major Depressive Disorder (MDD) were selected for quantitative analysis. Specifically, 5 studies were considered for the IVW with fixed effects approach [42-46], 7 for the MR-Egger regression [42-48], and 7 for the Weighted Median approach [42-48].

#### 3.2.1 IVW (Inverse-Variance Weighted)

The meta-analyses of studies that used the IVW method revealed high heterogeneity (*P*= 0.022; tau2= 0.008; I2= 65.1%; 95% CI: [8.6%; 86.7%]).

**Fig. (3):**
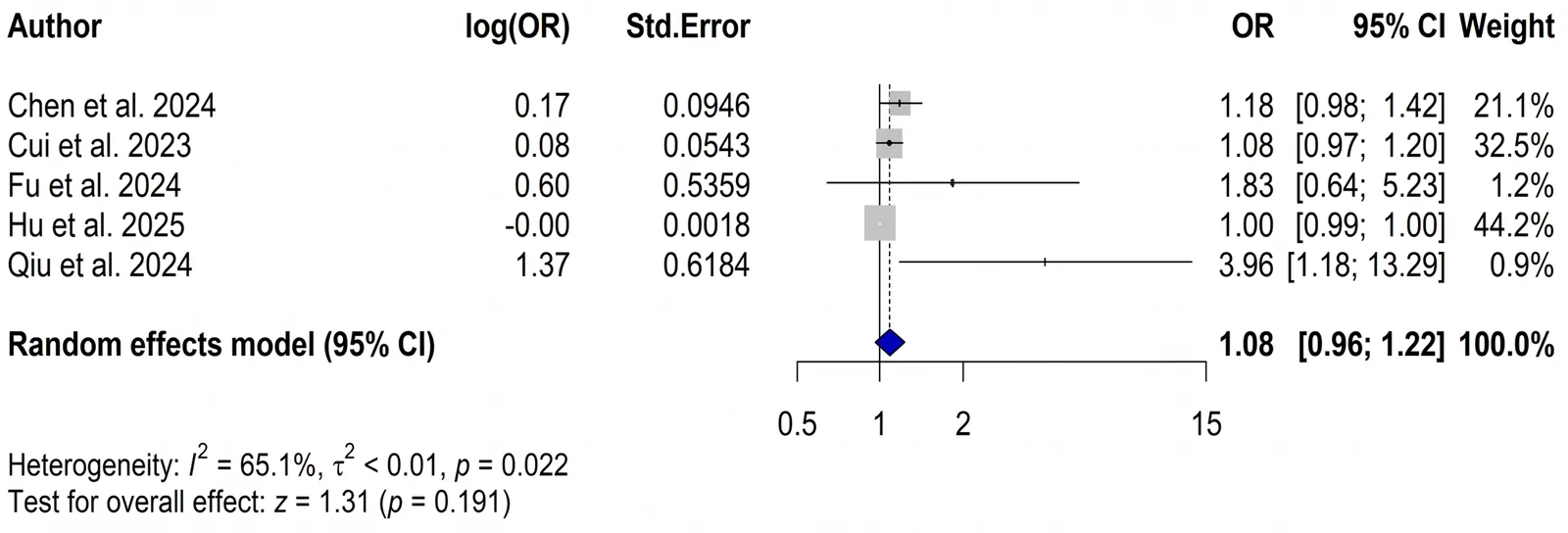
Forest Plot for MDD that used the MR-IVW method. MR estimates of the association between MDD and cancer using the MR-IVW method.

The forest plot (Fig. **3**) indicated no significant causal effect between cancer and MDD (OR= 1.08; 95% CI: [0.96; 1.^21^]; *P*= 0.191). The Funnel Plot (Supplementary File **2**) showed evidence of publication bias, a hypothesis confirmed by Egger's test (β = 1.67; 95% CI = [1.21; 2.^13^]; *P*= 0.006). However, the relatively small number of studies included limited the statistical power to detect such bias.

Supplementary File **2**: Contour-enhanced Funnel Plot for MDD that used the MR-IVW method.

#### 3.2.2 MR-Egger

**Fig. (4):**
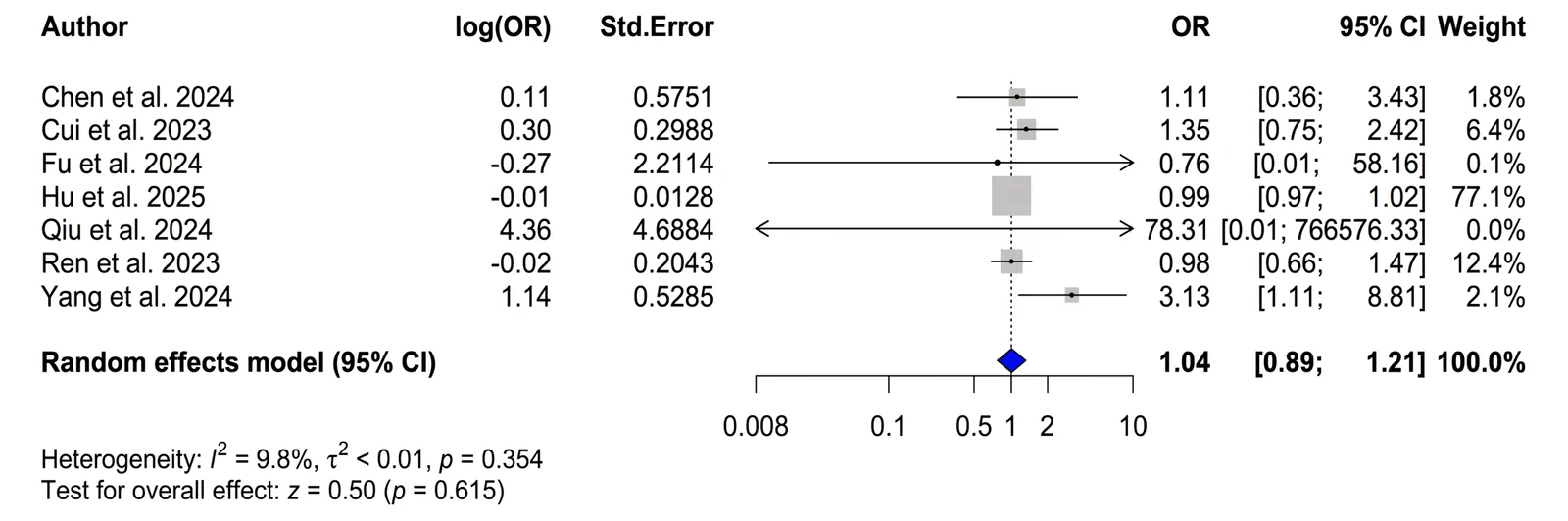
Forest plot for MDD that used the MR-Egger method. MR estimates of the association between MDD and cancer using the MR-Egger method.

Subsequently, we meta-analyzed the studies that used the MR-Egger method to assess whether the results supported or contradicted those obtained with the IVW method. In this case, heterogeneity among studies was not significant (*P*= 0.354; tau^2^ = 0.008; I^2^= 9.8%, 95% CI: [0.0%; 73.7%]). Furthermore, no causal effect between cancer and MDD was observed (OR = 1.04; 95% CI: [0.89; 1.^21^]; *P*= 0.615), as shown in the Forest Plot (Fig. **4**). The Funnel Plot (Supplementary File **3**) and Egger's test (β= 0.71; 95% CI= [0.0; 1.^43^]; *P*= 0.109) ruled out publication bias.

Supplementary File **3**: Contour-enhanced Funnel Plot for MDD that used the MR-Egger method.

#### 3.2.3 MR- Weighted Median

**Fig. (5):**
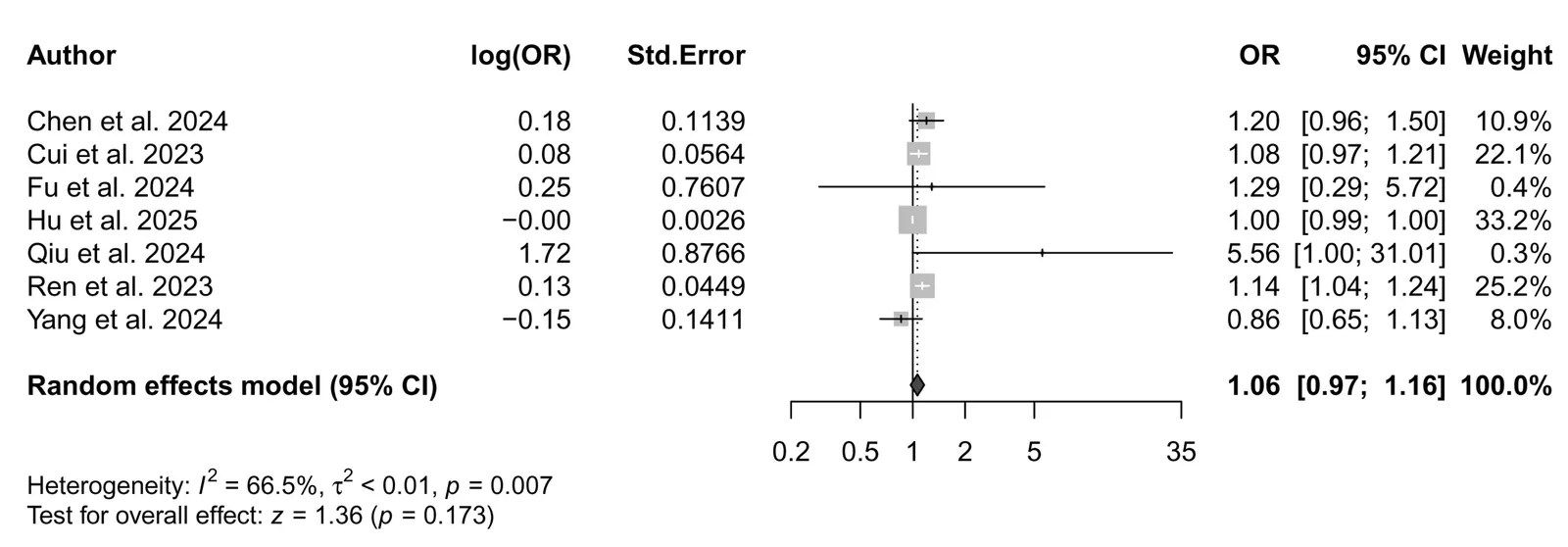
Forest plot for MDD that used the MR-Weighted Median approach. MR estimates of associations between MDD and cancer thanks to the MR-Weighted Median approach.

Finally, we meta-analyzed studies that used the MR-Weighted Median approach. In this case, heterogeneity among studies was significant (*P*= 0.007; tau^2^= 0.006; I^2^= 66.5%, 95% CI: [25.1%; 85%]), similar to the IVW approach. Again, no causal effect between cancer and MDD was observed (OR = 1.06; 95% CI: [0.97; 1.^16^]; *P*= 0.173), as shown in the Forest Plot (Fig. **5**). Both the funnel plot (Supplementary File **4**) and Egger's test (β= 1.20; 95% CI = [0.06; 2.^34^]; *P*= 0.095) ruled out publication bias.

Supplementary File **4**: Contour-enhanced Funnel Plot for MDD that used the MR-Weighted Median approach.

### 3.3 Schizophrenia

A total of 7 studies investigating schizophrenia were included in the quantitative analysis. Specifically, six studies were used for the Inverse-Variance Weighted (IVW, fixed effects) method [42-46, 48], seven for the MR-Egger regression [42-48], and seven for the Weighted Median method [42-48].

#### 3.3.1 IVW (Inverse-Variance Weighted)

**Fig. (6):**
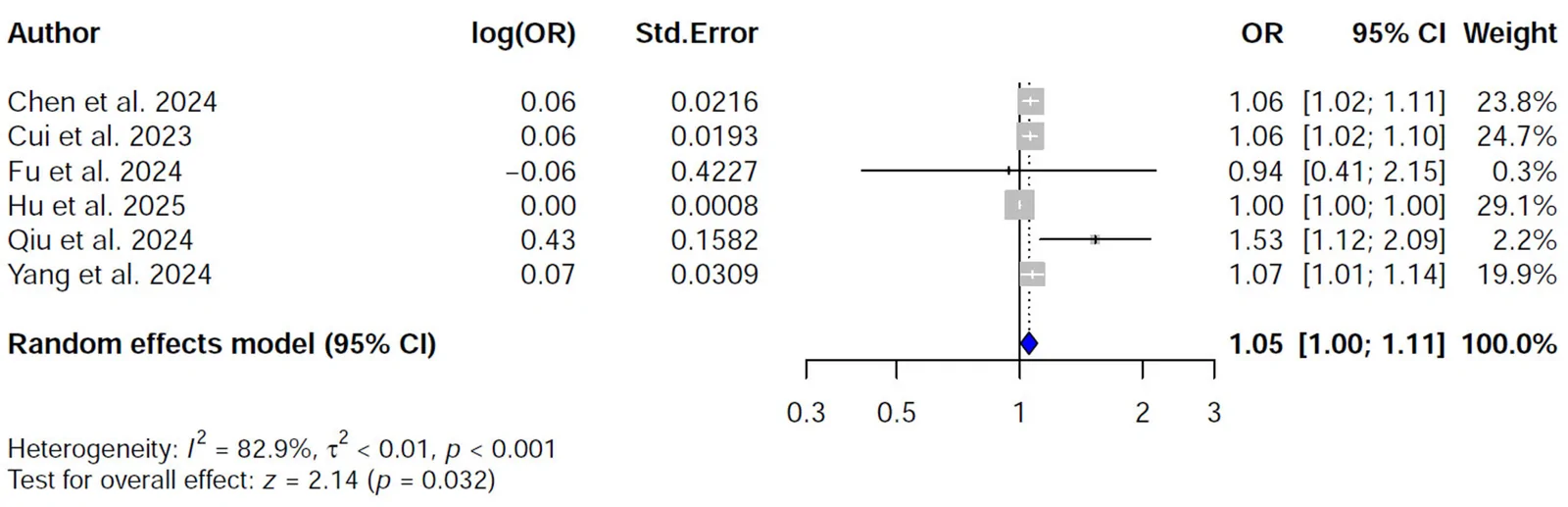
Forest Plot for schizophrenia that used the MR-IVW method. MR estimates of the association between schizophrenia and cancer, using the MR-Weighted Median approach.

Meta-analysis using the IVW method revealed high heterogeneity across studies (*P*< 0.001; tau2= 0.002; I2= 82.9%; 95% CI: [64%; 91.9%]). As shown in the Forest Plot (Fig. **6**), there was a statistically significant causal relationship between cancer and schizophrenia (OR = 1.05; 95% CI: [1.004; 1.105]; *P*= 0.032). The Funnel Plot (Supplementary File 5) indicated potential publication bias, a finding further supported by Egger's test (β = 2.16; 95% CI = [0.97; 3.^34^]; *P*= 0.024). However, the limited number of included studies may reduce the statistical power to detect such bias.

Supplementary File **5**: Contour-enhanced Funnel Plot for schizophrenia that used the MR-IVW method.

#### 3.3.2 MR-Egger

**Fig. (7):**
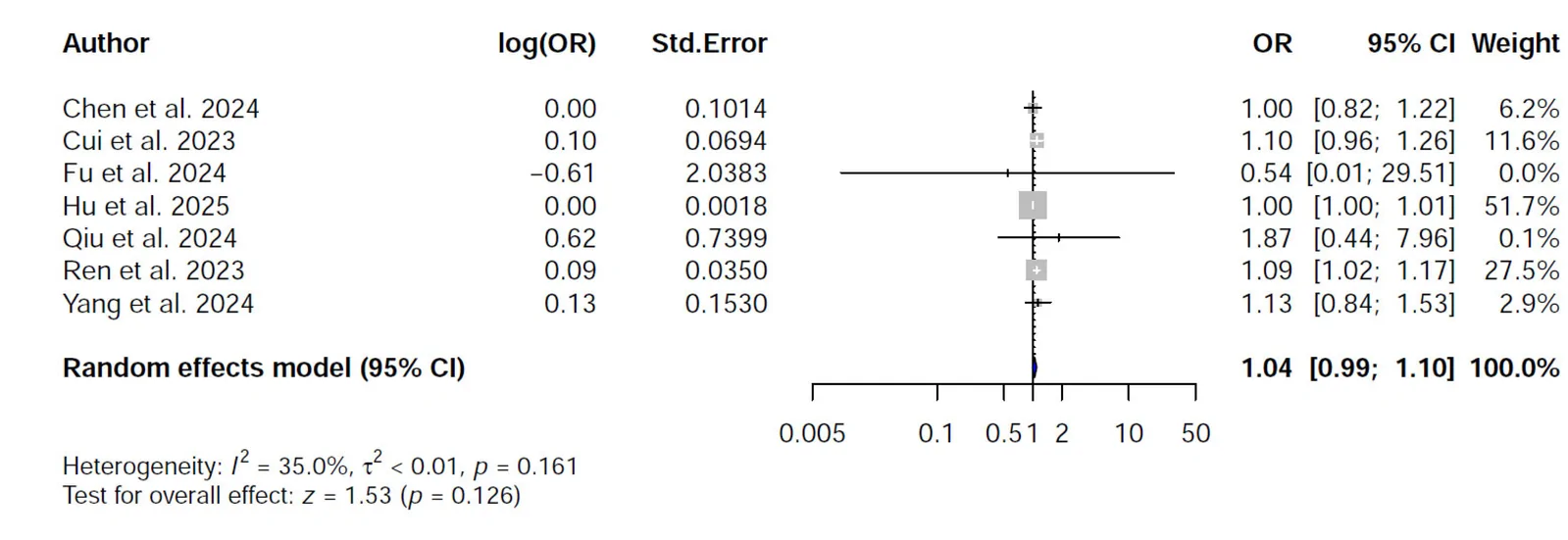
Forest Plot for schizophrenia that used the MR-Egger method. MR estimates of associations between schizophrenia and cancer, using the MR-Egger method.

Subsequently, we meta-analyzed studies that used the MR-Egger method to assess whether the results supported or contradicted those obtained with the IVW method. In this case, heterogeneity among studies was not statistically significant (*P*= 0.161; tau^2^ = 0.001; I^2^= 35%, 95% CI: [0.0%; 72.5%]). No significant causal relationship between cancer and schizophrenia was observed (OR= 1.04; 95% CI: [0.99; 1.0^9^]; *P*= 0.126), as illustrated in the forest plot (Fig. **7**). Neither the Funnel Plot (Supplementary File **6**) nor Egger's test (β= 0.85; 95% CI= [0.04; 1.^67^]; *P*= 0.096) provided evidence of publication bias.

Supplementary File **6**: Contour-enhanced Funnel Plot for schizophrenia that used the MR-Egger method.

#### 3.3.3 MR-Weighted Median

**Fig. (8):**
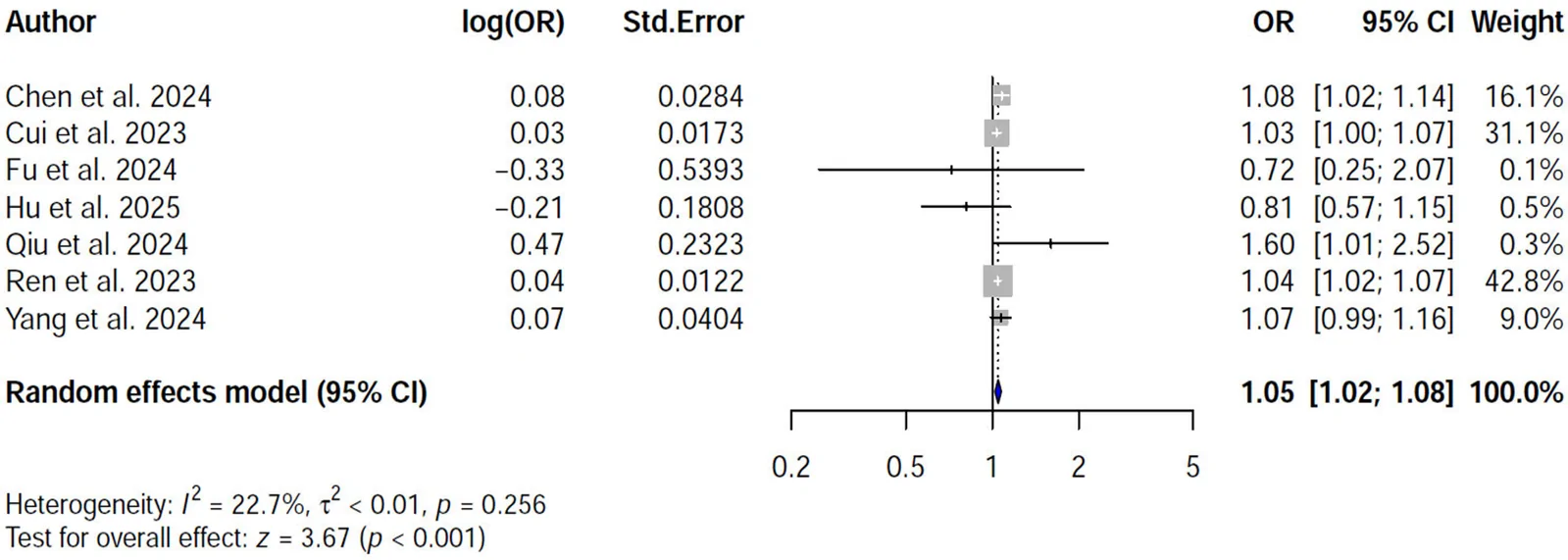
Forest Plot for schizophrenia that used the MR-Egger method. MR estimates of associations between schizophrenia and cancer, using the MR-Egger method.

Finally, a meta-analysis was conducted that included studies that used the MR-Weighted Median method. Heterogeneity among studies was not significant (*P*= 0.256; tau^2^= 0.0002; I^2^= 22.7%; 95% CI: [0.0%; 65.6%]), a result consistent with findings from the IVW method. A significant causal association between cancer and schizophrenia was observed (OR = 1.05; 95% CI: [1.02; 1.0^8^]; *P*= 0.0002), as shown in the Forest Plot (Fig. **8**). The Funnel Plot (Supplementary File **7**) and Egger's test (β= 0.17; 95% CI = [-1.18; 1.^51^]; *P*= 0.819) did not indicate any publication bias.

Supplementary File **7**: Contour-enhanced Funnel Plot for schizophrenia that used the MR-Weighted Median method.

### 3.4 Bipolar Disorder

A total of seven studies examining bipolar disorder were included in the quantitative analysis. Of these, six were considered for the Inverse-Variance Weighted (IVW, fixed effects) method [42-46, 48], seven for MR-Egger regression [42-48], and seven for the Weighted Median method [42-48].

#### 3.4.1 IVW (Inverse-Variance Weighted)

The meta-analyses conducted on the studies that used the IVW method revealed no significant heterogeneity across the included studies (*P*= 0.388; tau^2^< 0.001; I^2^= 4.5%, 95% CI: [0.0%; 75.8%]).

**Fig. (9):**
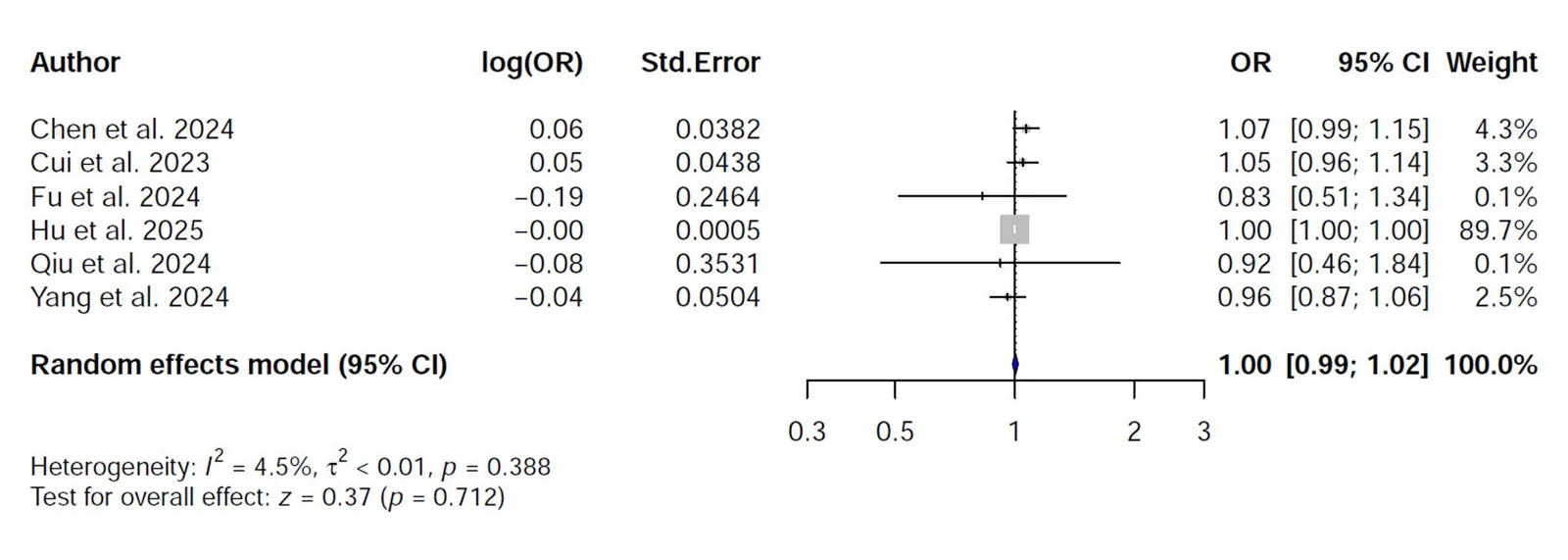
Forest Plot for bipolar disorder with an MR-IVW approach. MR estimates of associations between bipolar disorder and cancer, using the MR-IVW approach.

As shown in the Forest Plot (Fig. **9**), no significant causal association was found between cancer and bipolar disorder (OR= 1.003; 95% CI: [0.99; 1.0^2^]; *P*= 0.712). The Funnel Plot (Supplementary File 8) indicated no publication bias, a conclusion supported by the results of Egger's test (β= 0.18; 95% CI= [-0.82; 1.^18^]; *P*= 0.741). Nevertheless, the small number of studies may limit the statistical power to detect such bias.

Supplementary File **8**: Contour-enhanced Funnel Plot for bipolar disorder with an MR-IVW approach.

#### 3.4.2 MR-Egger

**Fig. (10):**
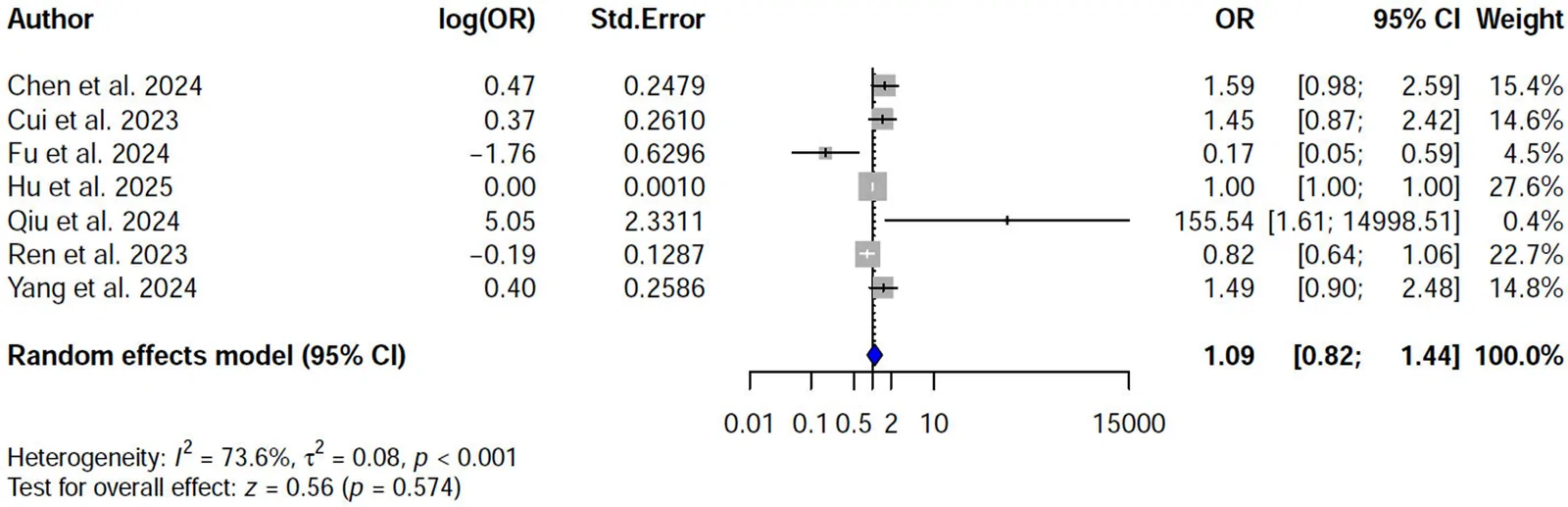
Forest Plot for bipolar disorder with an MR-Egger approach. MR estimates of associations between bipolar disorder and cancer, using the MR-Egger approach.

Subsequently, we performed a meta-analysis of studies that used the MR-Egger method to evaluate whether its results aligned with or diverged from those obtained through the IVW method. In this analysis, high heterogeneity was observed (*P*< 0.001; tau^2^ = 0.08; I^2^= 73.6%, 95% CI: [43.5%; 87.7%]). No significant causal relationship between cancer and bipolar disorder was detected (OR= 1.09; 95% CI: [0.82; 1.^21^]; *P*= 0.574), as illustrated in the Forest Plot (Fig. **10**). Likewise, neither Funnel Plot (Supplementary File **9**) nor Egger's test (β= 0.45; 95% CI= [-1.21; 2.^12^]; *P*= 0.618) provided evidence of publication bias.

Supplementary File **9**: Contour-enhanced Funnel Plot for bipolar disorder with a MR-Egger approach.

#### 3.4.3 MR- Weighted Median

**Fig. (11):**
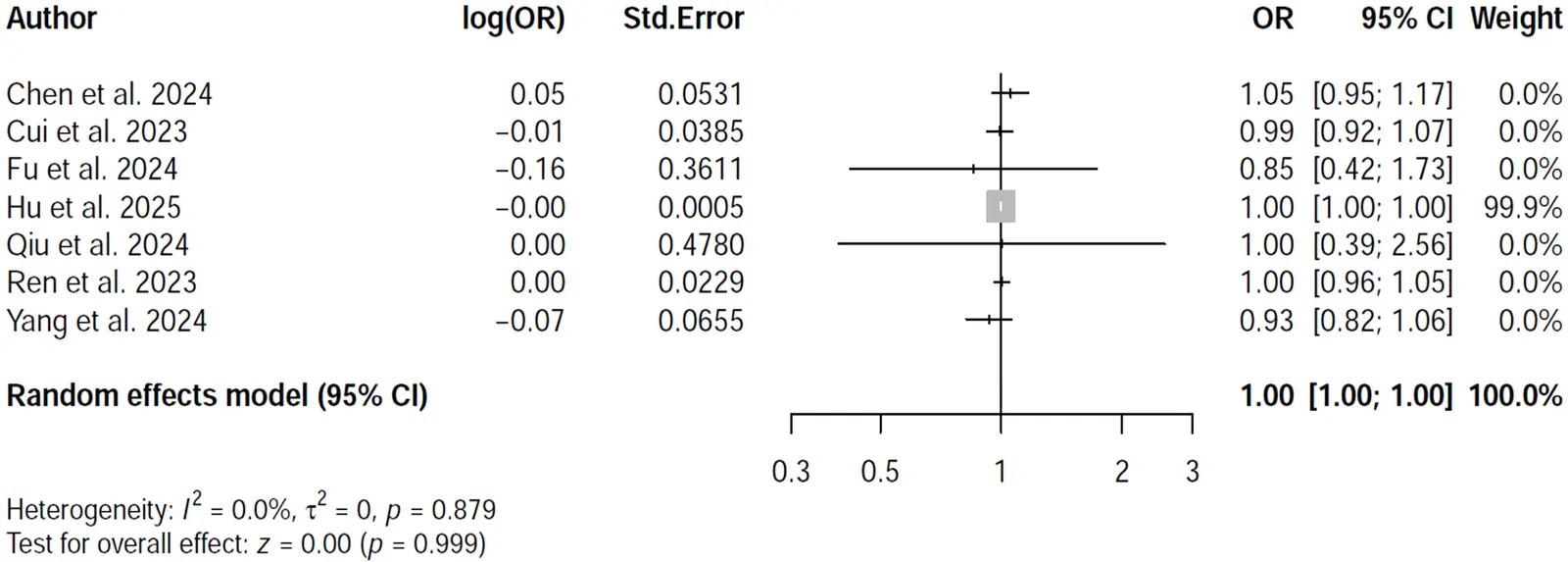
Forest Plot for bipolar disorder with a MR-Weighted Median method. MR estimates of associations between bipolar disorder and cancer, using the MR-Weighted Median method.

Finally, the studies that used the MR-Weighted Median method were meta-analyzed. In this case, heterogeneity was low and not significant (*P*=0.879; tau^2^=0; I^2^=0.0%; 95% CI: [0.0%; 70.8%]), consistent with the IVW findings. No evidence of a causal relationship between cancer and bipolar disorder was found (OR= 1; 95% CI: [0.999; 1.00^1^]; *P*=0.999), as shown in the Forest Plot (Fig. **11**). Both the Funnel Plot (Supplementary File **10**) and Egger's test (β= -0.09; 95% CI = [-0.65; 0.^46^]; *P*= 0.757) showed no indication of publication bias.

Supplementary File **10**: Contour-enhanced Funnel Plot for bipolar disorder with a MR-Weighted Median method.

## 4 DISCUSSION

The present systematic review and meta-analysis aimed to explore any associations between SPDs and cancer diseases by meta-analyzing MR studies available in the current literature.

Our findings highlighted a statistically significant association between cancer and SCZ using the IVW method (OR = 1.05; 95% CI: [1.004; 1.105]; *P*= 0.032). Conversely, using the other two MR methods, such as the MR-Egger method and the MR-Weighted Median, no significant associations were observed, also in SCZ.

In this regard, previous literature has investigated cancer risk in patients with SCZ [49]. However, there was also evidence suggesting a decreased cancer risk [50-53], and conversely, an increased one [54-56], especially among patients suffering from SCZ. In addition, some reports suggested decreased rates among first-degree relatives of SCZ patients [57], also due to genetic factors associated with SCZ that could protect against cancer. On the other hand, results suggesting an increased rate between cancer and SCZ referred to several risk factors which could increase cancer risk, such as obesity, diabetes, low physical activity level, high tobacco use, alcohol consumption, and antipsychotic medication treatment [58, 59]. However, further studies found no association between cancer risk among SCZ patients compared with the general population [60, 61]. Additionally, there were very few studies concerning specific solid tumors and their incidence rates among age subgroups in SCZ patients. For example, among lung cancer patients suffering from SCZ, both a higher [62, 63] and lower [64, 65] risk was observed, compared to the general population. Moreover, lower incidence rates for specific cancer subtypes could be linked to less adherence to screenings, as in the case of prostate cancer, which often endures undetected for a long period of time [66]. As regards Breast Cancer (BC), a systematic review found that women suffering from SCZ received less than 50% of the recommended screening compared to women without SCZ [67], highlighting the negative impact of SPDs in this setting [68]. This aspect could represent an approach that obscured care gaps specific to women with serious mental illness. In fact, SCZ women could deny cancer symptomatology, and BC diagnosis could occur in high-stage disease [69]. After diagnosis, women suffering from SPD did not receive the same cancer care compared to women without SPD due to their lower awareness and comprehension of the disease and consequent reduced cooperation with medical staff [70, 71].

BC was positively associated with smoking, drinking, obesity, and reduced physical activity [72], which were identified as potential risk factors in SPD populations, and previous evidence suggested a putative correlation between antipsychotic medications, increased prolactin, and breast tumors [73]. However, these correlations remain to be validated [74].

Moreover, several reports on BC highlighted significant associations with SCZ. In fact, it was assessed that the odds of BC increased by 56% with more than five years of antipsychotic use compared to non-consumption in SCZ women [75]. Rahman *et al*. (2022) [76] highlighted that women taking antipsychotics with prolactin-inducing properties were exposed to an increased risk of BC. In addition, another meta-analysis of observational studies suggested a moderate association between overall antipsychotic use and BC [77]. This result was probably due to hyperprolactinemia induction and was likely linked to an increase in metabolic side effects [78, 79], which represented a potential risk factor for BC [80]. On the other hand, previous reports evidenced that patients suffering from SCZ most of the time had less BC screening than the general population [67], with also lower related treatments [81], which inevitably led to higher mortality rates for these patients. Surely, the association evidenced between SPDs and cancer represented a hot topic that could be considered with all confounders present in the current disorganized epidemiology. Adopting the MR method to assess causal associations could balance confounding bias in observational studies.

The present study had several limitations. First of all, the current study did not perform new MR analyses from raw GWAS data, while it aggregated previously published MR results. Data obtained from the present systematic review and meta-analysis, particularly regarding a possible schizophrenia-cancer association, should be interpreted cautiously, given inconsistencies across MR methods, high heterogeneity, and the small number of available studies. Secondly, important MR quality checks, such as F-statistics for instrument strength, clumping procedures, pleiotropy correction (*e.g.*, MR-PRESSO), and harmonization issues, could not be assessed. The schizophrenia–cancer association appeared significant under IVW, but not under MR-Egger, an inconsistency strongly suggesting that the result could be sensitive to pleiotropy or weak instruments.

## CONCLUSION

The literature on this topic is scarce, and further studies are needed to better assess the association between SPDs and cancer. Thus, our results should be interpreted cautiously due to inconsistencies across MR methods, high heterogeneity, and the limited number of available studies.

Surely, some behaviors in patients with SPDs were identified as risk factors for several cancer types, like obesity, childlessness, and use of anti-SPDs drugs, due to the increased level of prolactin. Thus, the need to improve health management strategies for individuals with SPDs remains unmet. Further investigation into biological functions and interdisciplinary associations will inform future interventions and public health policies to moderate cancer risk in this susceptible population [82].

## Figures and Tables

**Table 1:** Study characteristics (n=7).

Study	**Severe Psychiatric Disease**	**Study Design**	**Outcome - Cancer Disease**	**Highlights**
Chen *et al*. 2024 [42]	BD MDD SCZ	MR study	Lung Carcinoma	SCZ represents a risk factor for lung carcinoma, with mediating factors such as smoking and alcohol consumption.
Cui *et al*. 2023 [43]	BD MDD SCZ	MR study	BC	SCZ improves susceptibility to BC associated smoking as a mediating factor.
Fu *et al*. 2024 [44]	BD MDD SCZ	MR study	CRC EG GC	There is a weak association between SPD and the risk of digestive tract cancers. PD seems to have a potential protective effect against CRC.
Hu *et al*. 2025 [45]	ADHD ASD BD MDD SCZ	MR study	BC	SCZ, BD, MDD, ADHD, and ASD are not causally linked to BC. AD reports a causal and positive relationship with BC.
Qiu *et al*. 2024 [46]	BD MDD SCZ	MR study	Thyroid Cancer	MDD and SCZ may be positively associated with thyroid cancer risk, while also revealing a correlation between BD and thyroid cancer.
Ren *et al*. 2023 [47]	BD MDD PTSD PD Obsessive-compulsive disorder SCZ	MR study	BC	SCZ and BC report false-positive results due to horizontal pleiotropy rather than a true cause-and-effect relationship.
Yang *et al*. 2024 [48]	ASD BD MDD PD SCZ	Bidirectional MR study	Glioma	An increased risk of non-GBM glioma in individuals with SCZ provides a possible insight into glioma etiology.

**Abbreviations:** AD: anxiety disorder; ADHD: attention-deficit/ hyperactivity disorder; ASD: autism spectrum disorder; BC: Breast Cancer; BD: Bipolar Disorder; MDD: Major Depressive Disorder; CRC: colorectal cancer; EC: esophagus cancer; GC: Gastric cancer; MR: Mendelian randomization; PD: Panic Disorder; PTSD: Post-Traumatic Stress Disorder; SCZ: Schizophrenia.

## Data Availability

All data generated or analyzed during this study are included in this published article.

## LIST OF ABBREVIATIONS

SCZ: Schizophrenia
BD: Bipolar Disorder
MDD: Major Depressive Disorder
SPD: Severe Psychiatric Disorders
MR: Mendelian Randomization
IVs: Instrumental Variables
IVW: Inverse-Variance Weighting

## STANDARDS OF REPORTING

PRISMA guidelines were followed.
